# Comparison of *Staphylococcus aureus* tolerance between antimicrobial blue light, levofloxacin, and rifampin

**DOI:** 10.3389/fmicb.2023.1158558

**Published:** 2023-05-25

**Authors:** Jemi Ong, Rose Godfrey, Alexa Nazarian, Joshua Tam, Brad M. Isaacson, Paul F. Pasquina, Dustin L. Williams

**Affiliations:** ^1^Department of Biomedical Engineering, University of Utah, Salt Lake City, UT, United States; ^2^Department of Orthopaedics, University of Utah, Salt Lake City, UT, United States; ^3^Wellman Center for Photomedicine, Massachusetts General Hospital, Boston, MA, United States; ^4^Department of Dermatology, Harvard Medical School, Boston, MA, United States; ^5^The Geneva Foundation, Tacoma, WA, United States; ^6^Department of Physical Medicine and Rehabilitation, The Musculoskeletal Injury Rehabilitation Research for Operational Readiness, Uniformed Services University, Bethesda, MD, United States; ^7^The Center for Rehabilitation Sciences Research, Uniformed Services University, Bethesda, MD, United States; ^8^Department of Rehabilitation, Walter Reed National Military Medical Center, Bethesda, MD, United States; ^9^Department of Pathology, University of Utah, Salt Lake City, UT, United States

**Keywords:** biofilms, osseointegration, blue light, antibiotics, infection

## Abstract

**Background:**

Bacterial biofilms readily develop on all medical implants, including percutaneous osseointegrated (OI) implants. With the growing rate of antibiotic resistance, exploring alternative options for managing biofilm-related infections is necessary. Antimicrobial blue light (aBL) is a unique therapy that can potentially manage biofilm-related infections at the skin-implant interface of OI implants. Antibiotics are known to have antimicrobial efficacy disparities between the planktonic and biofilm bacterial phenotypes, but it is unknown if this characteristic also pertains to aBL. In response, we developed experiments to explore this aspect of aBL therapy.

**Methods:**

We determined minimum bactericidal concentrations (MBCs) and antibiofilm efficacies for aBL, levofloxacin, and rifampin against *Staphylococcus aureus* ATCC 6538 planktonic and biofilm bacteria. Using student *t*-tests (*p* < 0.05), we compared the efficacy profiles between the planktonic and biofilm states for the three independent treatments and a levofloxacin + rifampin combination. Additionally, we compared antimicrobial efficacy patterns for levofloxacin and aBL against biofilms as dosages increased.

**Results:**

aBL had the most significant efficacy disparity between the planktonic and biofilm phenotypes (a 2.5 log_10_ unit difference). However, further testing against biofilms revealed that aBL had a positive correlation between increasing efficacy and exposure time, while levofloxacin encountered a plateau. While aBL efficacy was affected the most by the biofilm phenotype, its antimicrobial efficacy did not reach a maximum.

**Discussion/conclusion:**

We determined that phenotype is an important characteristic to consider when determining aBL parameters for treating OI implant infections. Future research would benefit from expanding these findings against clinical *S. aureus* isolates and other bacterial strains, as well as the safety of long aBL exposures on human cells.

## 1. Introduction

Bacterial biofilms readily develop on all medical implants. Percutaneous devices, such as transfemoral osseointegrated (OI) implants, are particularly susceptible to biofilm formation as they provide a trifecta conducive to this process: an air, liquid, and solid interface (Green, 1983, 1984). While OI implant technology is becoming increasingly adopted, the skin-implant interface remains vulnerable. Among five available OI implant systems, all are susceptible to soft tissue infection from bacteria on the skin, mucosa, or implant surface (Izadi et al., 2019). The rate of soft-tissue infection soars as high as 75%, although most cases can be effectively treated with systemic antibiotics (Izadi et al., 2019). Nevertheless, the growing threat of antibiotic resistance challenges this line of defense. As OI implant technology becomes more widely adopted, we seek to develop alternative strategies to manage OI implant-related infections, notably those underscored by biofilm formation.

Antimicrobial blue light (aBL) is a potential alternative for managing biofilm burden. It is a localized therapy with low toxicity against skin cells (Joshua et al., 2020). Additionally, light does not target a specific mechanism of cellular function, and bacteria are unlikely to develop resistance against aBL (Yin et al., 2013). We are developing a specialized aBL unit that may be docked onto the percutaneous post of an OI implant and administer aBL to the skin-implant interface. The objective is to prevent bacterial division and mitigate biofilm load present on the device surface or superficial regions of the soft tissue surrounding the implant. While this approach is promising, a pivotal point to consider in device parameter development is the differences in efficacy aBL might have on planktonic vs. biofilm bacteria, which will exist in unknown proportions at the skin-implant interface.

It is generally accepted that biofilms exhibit greater antibiotic tolerance than planktonic bacteria. Importantly, it is unknown if this pattern also holds for aBL therapy. Antibiotic efficacy is often defined by a minimum inhibitory concentration (MIC), the concentration at which an antibiotic prevents planktonic bacterial division in a broth solution (MIC values usually range from 0.125 to 2 μg/ml) (Murillo et al., 2006, 2008; Schuurmans et al., 2009; Asseray et al., 2016). Another related value is the minimum bactericidal concentration (MBC), often described as the concentration at which an antibiotic reduces the viability of planktonic bacteria by a pre-determined amount (typically 3-log_10_ colony forming units (CFU), or 99.9% reduction). MBC values are frequently 2x-10x higher than MIC values (Koljalg et al., 2002; Biedenbach et al., 2007; Tato et al., 2014). However, data commonly show that antibiotics must be dosed orders of magnitude above planktonic MIC or MBC values to affect biofilms (Mandell et al., 2019). Bacteria in a biofilm naturally tolerate high antibiotic concentrations due to characteristics such as high cellular density and extracellular polymeric substances that bind and neutralize antibiotics (Walters et al., 2003; Mulcahy et al., 2008). Biofilm cells also have lower metabolic activity, decreasing certain antibiotics' efficacy (Gilbert et al., 1990). Additionally, water channels may form through which antibiotics can be diffused and miss their target altogether (Uruén et al., 2019). As a result, available MIC/MBC values against planktonic bacteria have little to no relevance against biofilms.

We established a methodology for comparing MBC values for two antibiotics against planktonic *Staphylococcus aureus* ATCC 6538 and its biofilm phenotype. *S. aureus* is one of the most common bacteria found on orthopedic implants (Lauderdale et al., 2010; Nandakumar et al., 2013), and our lab has had years of experience growing consistent, robust biofilms with *S. aureus* ATCC 6538. As the end goal was to use aBL in OI implant applications, antibiotics common in orthopedic practice were chosen: levofloxacin and rifampin (Muller-Serieys et al., 2009; Guillaume et al., 2012). Both interfere with specific, essential metabolic processes to disrupt DNA replication, resulting in fatal downstream effects (Davis and Bryson, 1994; Zimmerli and Sendi, 2019). As aBL excites endogenous porphyrins to generate harmful reactive oxygen species (ROS), the metabolic state of planktonic or biofilm bacteria is unlikely to affect its antimicrobial efficacy (Wang et al., 2017). We hypothesized that aBL would exhibit a lesser antimicrobial efficacy discrepancy between the two bacterial phenotypes than those observed with levofloxacin and rifampin.

To compare disparity profiles, we first established a baseline CFU concentration for planktonic suspensions and CDC reactor-grown biofilms using *S. aureus* ATCC 6538. MBCs, pre-determined as a 3-log_10_ unit reduction, were established for individual treatments of levofloxacin and rifampin against both bacterial phenotypes. MBCs were determined for a combination therapy of levofloxacin + rifampin against planktonic and biofilm bacteria. Similarly, by adjusting the irradiation parameters of our aBL device, we evaluated the energy required to reduce planktonic suspensions and biofilms of *S. aureus* by 3-log_10_ units. MBC data across all groups were compared, particularly the percent increase between the planktonic and biofilm states. These data provided insight into aBL device development and treatment design. They exposed a facet of aBL research that should be expanded to develop aBL therapy as an antibiotic alternative. Our research has provided a stepping stone toward the goal of using aBL in clinical practice and the more widespread use of OI implants.

## 2. Materials and methods

### 2.1. Supplies, instruments, and reagents

Tryptic soy broth (TSB) was purchased from MilliporeSigma (Burlington, MA), Petri dishes, agar, and general supplies/reagents from Fisher Scientific (Hampton, NH), and brain heart infusion (BHI) broth from Research Products International (Mt Prospect, IL). CDC biofilm reactors and titanium (Ti) coupons were purchased from Biosurface Technologies (Bozeman, MT). Levofloxacin was acquired from Chem-Impex International (Wood Dale, IL) and rifampin from ThermoFisher Scientific (Waltham, MA).

### 2.2. Biofilm growth and baseline bacterial concentration

Biofilms of *S. aureus* ATCC 6538 were grown on Ti coupons in a CDC biofilm reactor (Ong et al., 2022). Coupons were pre-treated to support biofilm growth: exposed to hydrochloric acid (HCl; 36.5–38%) for 24 h, followed by a 10 min DI water rinse and 10 min of DI water sonication. HCl treatment roughened the coupon surface to improve biofilm adherence. This process was performed every 6–8 reactor cycles as the surfaces smoothed out with frequent cleaning. Additionally, coupons were treated with nitric acid before each growth cycle (60% nitric acid for 30 min, followed by a 10 min DI water rinse and 10 min DI water sonication; slightly modified ASTM Standard B 600-91). This step removed remaining post-cleaning surface contaminants that could impede biofilm growth and adherence.

Coupons were placed in the holding arms of a CDC biofilm reactor, and masking tape was used to cover the inner-facing surface of each coupon. The assembled reactor was sterilized, filled with 500 ml of 100% BHI, and inoculated with 1 ml of an *S. aureus* 0.5 McFarland solution. The reactor was placed on a hot stir plate at 37°C and 130 rpm. Biofilms grew on the unmasked side of the coupon over a 72 h growth period: 24 h 100% BHI broth batch phase, 24 h 20% BHI broth flow, and 24 h 10% BHI broth flow, with a flow rate of 6.9 ml/min. After 72 h of growth, reactor arms were briefly immersed in sterile PBS to knock off loosely adherent cells. Masking tape was aseptically removed, and coupons were quantified in 2 ml of PBS using a 10-fold dilution series and plated on TSB agar. The CFU counts of 16 coupons were averaged, and the resulting value was utilized as the baseline bacterial concentration for all subsequent tests (~10^9^).

### 2.3. Comparable planktonic bacterial suspensions of 10^9^ CFU/ml

Biofilms on Ti coupons produced ~10^9^ CFU/coupon. We formulated planktonic suspensions that were ~10^9^ CFU/ml to make accurate comparisons. The required turbidity to achieve a 10^9^ CFU/ml was experimentally determined using a colorimeter. More specifically, *S. aureus* colonies from a fresh overnight culture were suspended in 10% BHI by vortexing for 1–2 min. By quantifying different percent turbidities in triplicate, it was determined that 8–9% turbidities correlated to approximately 10^9^ CFU/ml.

We chose to test the bacteria in lag phase (phase present immediately after suspension), although MBC testing against log or stationary phase is more common (Murillo et al., 2006, 2008; Saginur et al., 2006; Trampuz et al., 2007). The rationale for this decision was that 10% BHI was utilized throughout the experiments as it had adequate transparency necessary for aBL treatments; growing bacteria to 10^9^ CFU/ml in 10% broth until log or stationary phase was attained would have exhausted the nutrient supply, and bacteria could have entered the first stages of biofilm formation (Legner et al., 2019). Alternatively, if bacteria were inoculated into 100% BHI, there would have been no way to regulate nutrient depletion during bacterial growth to match the 10% BHI concentration used in the biofilm experiments.

### 2.4. Levofloxacin and rifampin

#### 2.4.1. Determining the MBC of levofloxacin and rifampin against planktonic bacteria

All 8–9% turbidity planktonic suspensions were vortexed for 1 min and sonicated for 10 min before adding antibiotics. A 100 μL antibiotic-free sample was quantified as baseline control. Stock solutions were added to 10 mL of the 8–9% turbidity planktonic suspensions (vortexed for 2 min) such that the final antibiotic concentrations matched reported MICs, 0.015 μg/ml for levofloxacin, and 0.12 μg/ml for rifampin (Murillo et al., 2006). A 1 ml aliquot of the mixture was pipetted into a sterile microcentrifuge tube; enough mixture was made so that this process could be repeated 16 times for *n* = 16 samples. Microcentrifuge tubes were placed in a shaker incubator (37°C, 60 rpm) for 24 h. Each sample was quantified, and the remaining CFU/ml after treatment was calculated (see Section 2.4.3). Using the MIC as a starting point, we increased the antibiotic concentrations by 2x - 10x until we attained a 3-log_10_ CFU reduction, or the antibiotic's MBC. Additionally, 16 samples of *S. aureus* without antibiotics were placed in the incubator for 24 h and quantified. These non-antibiotic controls helped ascertain if significant cellular death or growth occurred during the incubation period independent of antibiotic action.

Following independent levofloxacin and rifampin experiments, the MBC for the levofloxacin + rifampin (L/R) combination therapy was similarly determined.

#### 2.4.2. Determining the MBC of levofloxacin and rifampin against biofilms

Biofilm coupons were placed into 50 ml conical tubes (biofilm face up, taped face down). Stock solutions containing the planktonic antibiotic MBC concentrations were made in sterile 10% BHI. Two ml of antibiotic solution were carefully added to each conical tube, taking care not to disturb the biofilms. Conical tubes were placed in the shaker incubator (60 rpm, 37°C) for 24 h, after which the coupons were quantified using the spin-down method (outlined in Section 2.4.3). Antibiotic concentrations were doubled until we found the biofilm MBC. Untreated biofilm coupons were also placed in 10% BHI, incubated for 24 h, and quantified. They helped determine if biofilm bacteria death occurred independent of antibiotic treatment.

#### 2.4.3. Spin down quantification

A spin-down method was used to remove the antibiotics from solution and halt further antibiotic action (prevented residual kill). For biofilm experiments, coupons were first vortexed for 1 min, placed in a sonicating water bath for 10 min, and vortexed again for 10 s. One ml of each suspension was transferred to a microcentrifuge tube.

Microcentrifuge tubes from biofilm and planktonic experiments were then centrifuged in an Eppendorf Centrifuge 5415 (Hamburg, Germany) for 3 min at 5,000 rpm. Centrifuging forced the bacterial cells to clump into a pellet while the antibiotic remained in solution. Without disturbing the pellet, 900 μL of solution was removed and replaced with 900 μL of sterile PBS, diluting the antibiotic concentration by one order of magnitude. The diluted solutions were vortexed for 2 min, resuspending the bacteria and any remaining antibiotic. This process was repeated until the antibiotic concentration was reduced to below the MIC (2–4 times). On the final dilution, 100 μL of small, sterile glass beads (~100 μm diameter) were added to each microcentrifuge tube before a 2 min vortex. The beads ensured thorough bacterial re-suspension. Microcentrifuge tubes were placed in a sonicating water bath for 10 min and quantified using a 10-fold dilution series.

### 2.5. aBL efficacy testing

We used an aBL prototype device developed for a related project to determine aBL efficacy (Ong et al., 2022). The device consisted of 12 LEDs in a circular array that emitted 405 nm light. The LEDs were angled (30°) inward, enabling even irradiation of cylindrical samples. The unit was run at 0.250 A, correlating to a 150 mW optical power output.

#### 2.5.1. Determining the MBC of aBL against planktonic bacteria

Prepared bacterial suspensions (see Section 2.3) were vortexed for 1 min and put in a sonicating water bath for 10 min. One hundred μl of the suspensions were removed and quantified as a control. Large glass tubes (15 × 150 mm) were filled with 2 ml of *S. aureus* solution and clamped such that the bottom of the tube was centered in the middle of the LED array and resided 1 cm above the LEDs. aBL exposure times were adjusted in 30–60 min increments until the MBC was determined, i.e., the length of aBL exposure at 150 mW optical power that resulted in a 3-log_10_ CFU reduction. All samples were quantified using a 10-fold dilution series.

#### 2.5.2. Determining the MBC of aBL against biofilms

Biofilm coupons were carefully placed into large glass tubes (15 × 150 mm; biofilm side face down, taped side up). A stock solution of 1.25 μg/ml nafcillin and 10% BHI was prepared beforehand, and 2 ml was carefully added to each tube. The rationale for adding nafcillin (1.25 μg/ml was 20x its MIC value) was that it prevented bacterial division during longer aBL exposures. At this concentration, nafcillin had minimal bactericidal activity (Section 2.6). Sterile loops were used to remove air pockets trapped between the coupon and tube wall, taking care not to disturb the biofilms. Tubes were centered 1 cm above the LEDs in the center of the array. Beginning with the planktonic MBC value, exposure times were increased in 30–60 min increments until a biofilm MBC was determined. Due to the addition of nafcillin, the spin-down method (Section 2.4.3) was utilized during quantification. Each exposure time was experimentally tested with *n* = 16 samples.

### 2.6. Effect of different initial CFU concentrations on antimicrobial efficacy

As a result of observations from the above experiments, additional tests were performed to investigate the effect of initial CFU concentration on antimicrobial efficacy. Bacterial suspensions of 10^6^, 10^7^, 10^8^, and 10^9^ CFU/ml were made. Planktonic MBC concentrations for levofloxacin, rifampin, and aBL were each tested in triplicate against all four starting concentrations. Additionally, the 20x MIC for nafcillin was tested to ensure it did not contribute to overall kill in the aBL experiments.

### 2.7. Statistical analyses

Student *t*-tests (*p* ≤ 0.05) were used for all statistical analyses. Tests were used to compare the efficacy of either levofloxacin, rifampin, L/R, or aBL against bacteria in the planktonic lag phase vs. the biofilm state. Comparisons across groups did not focus on CFU count but the fold-increase in the dosage required to attain a 3-log_10_ CFU reduction. Subsequently, student *t*-tests were not performed between groups.

## 3. Results

### 3.1. MBC results for levofloxacin, rifampin, levofloxacin + rifampin, and aBL against planktonic bacteria

Planktonic MBCs were determined for levofloxacin, levofloxacin + rifampin (L/R), and aBL (see Table 1). Twenty-four-hour planktonic controls showed a 0.1–0.5 log_10_ reduction independent of antimicrobial treatment. Rifampin reached its solubility limit in 10% BHI before attaining an approximately 3-log_10_ CFU reduction. Subsequently, rifampin's solubility limit at 0.48 mg/ml was utilized for comparison purposes.

**Table 1:** Representation of the MBC values for aBL, levofloxacin, rifampin, and levofloxacin+rifampin.

	**aBL (+ nafcillin)**	**Levofloxacin (mg/ml)**	**Rifampin (mg/ml)**	**Levofloxacin + Rifampin (mg/ml)**
Planktonic MBC	2.5 h (+1.25 μg/ml)	2.4	>0.48	2.4 + 0.48
Biofilm MBC	10 h (+1.25 μg/ml)	9.6	>0.48	1.2 + 0.24

#### 3.1.1. Effect of planktonic MBC values on biofilms

**Figure 1:**
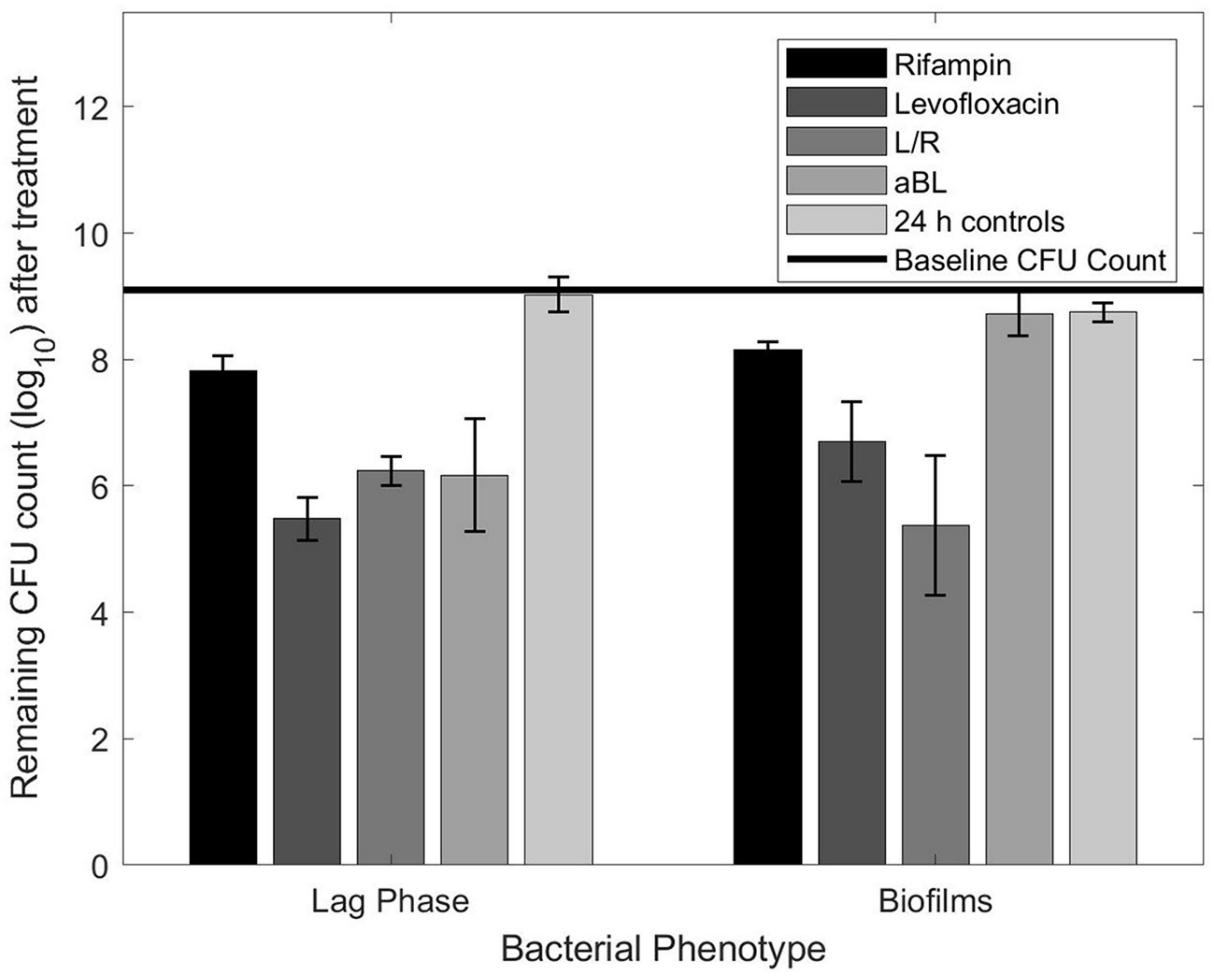
Remaining CFU count obtained after exposing planktonic or biofilm bacteria to planktonic MBCs listed in Table 1.

Figure 1 shows the results of using the planktonic MBC values against biofilms of the same starting CFU concentration. Unlike the planktonic controls, the biofilms had a slight loss in vitality after 24 h in the incubator (~0.5 log_10_ reduction). Levofloxacin (at 2.4 mg/ml) reduced biofilms by 1.8-log_10_ units, equating to a 1-log_10_ efficacy disparity between the lag phase planktonic bacteria and biofilms (statistically significant with a student *t*-test; *p* ≤ 0.05). Additionally, both rifampin and L/R results were statistically significant (student *t*-test, *p* ≤ 0.05), although with more minimal efficacy disparities. Rifampin had a 0.33 log_10_ difference while L/R increased in efficacy against biofilms by 0.86 log_10_ (Figure 1). Lastly, a 2.5 h exposure of aBL resulted in a 0.6 log_10_ CFU reduction, which was similar to reductions observed in the control samples (Figure 1). The aBL planktonic MBC had no observable effect against biofilms, and the resulting 2.5 log_10_ difference in efficacy was statistically significant with a student *t*-test; *p* ≤ 0.05.

### 3.2. MBC results for levofloxacin, rifampin, levofloxacin + rifampin, and aBL against biofilms

Both levofloxacin and aBL had significant dosage increases between the biofilm and planktonic MBCs (Table 1). L/R was unique in that it experienced a decrease between the biofilm and planktonic MBC (Table 1). Baseline controls taken during the aBL experiments showed that nafcillin did not have substantial bactericidal side effects during aBL treatments. A biofilm MBC for rifampin was not determined as the solubility limit had been met during planktonic experiments.

**Figure 2:**
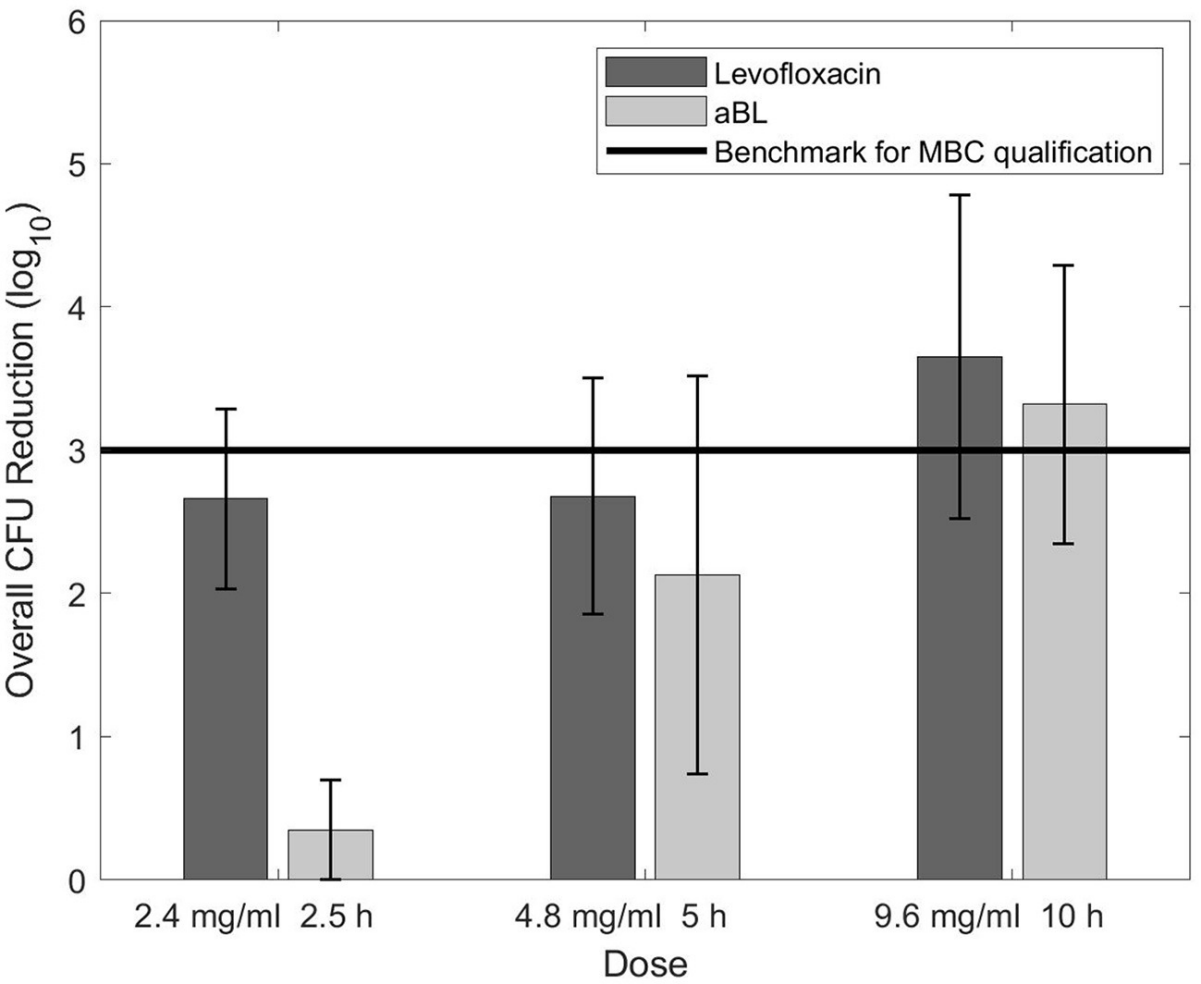
CFU reduction as the result of increasing dose/exposure time for levofloxacin and aBL.

Figure 2 shows data obtained while determining the biofilm MBC for levofloxacin and aBL. Levofloxacin had no change in CFU reduction with the first increase in dosage (2.4–4.8 mg/ml), yet a noticeable difference when the dose was adjusted to 9.6 mg/ml. In contrast, aBL demonstrated a positive trend in CFU reduction during each increase in aBL exposure. Efforts to test antibiotic concentrations past 9.6 mg/mL did not succeed as levofloxacin reached its solubility limit in 10% BHI.

### 3.3. Effect of different starting CFU concentrations on antimicrobial efficacy

**Figure 3:**
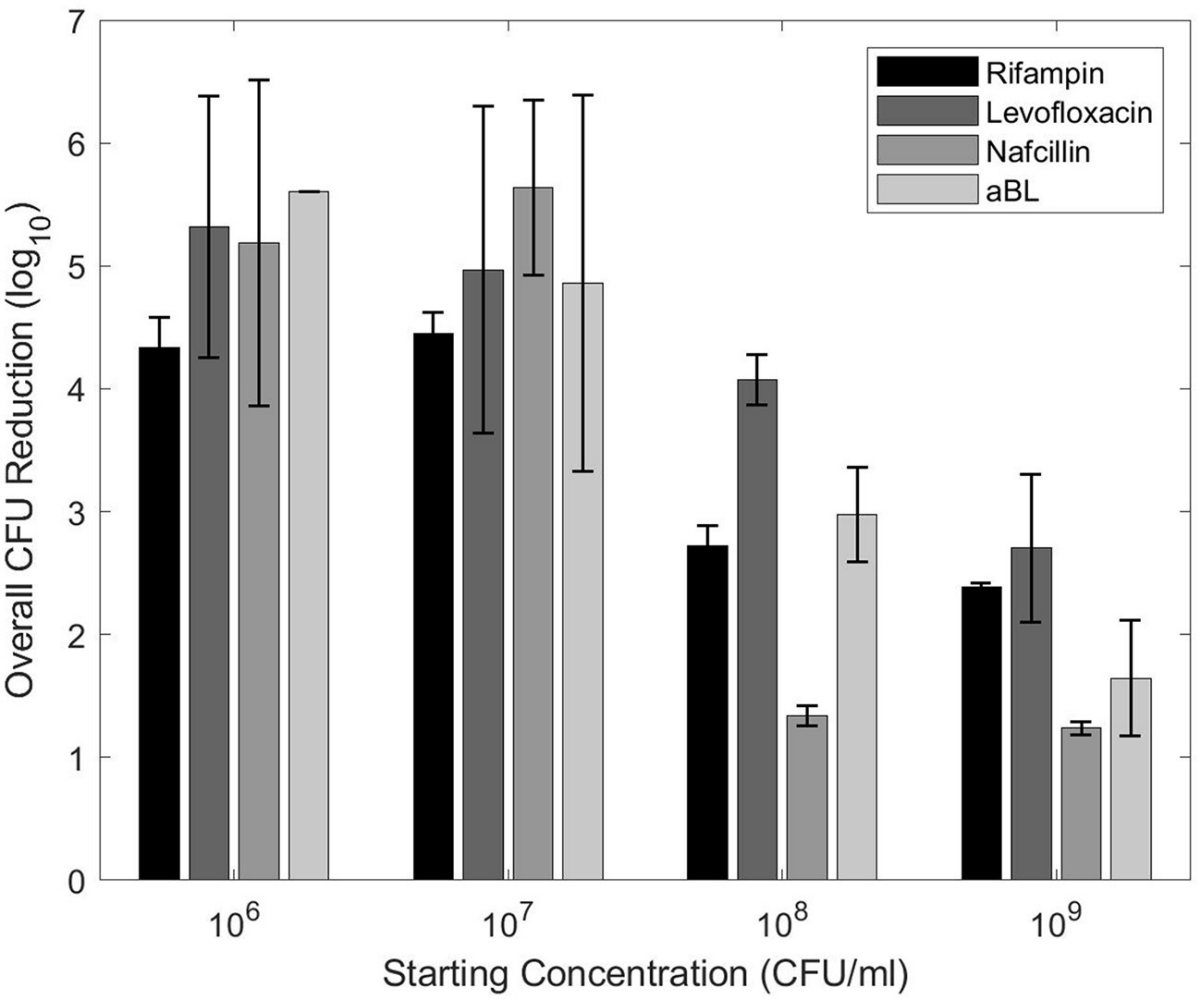
CFU reductions for each treatment with different planktonic starting concentrations. The planktonic MBCs from Table 1 were utilized.

The planktonic antibiotic MBCs were orders of magnitude larger than MBCs found in published literature (Campion et al., 2005; Murillo et al., 2006; Zimmerli and Sendi, 2019) (Figure 3). The aBL MBC steadily decreased in overall CFU reduction as starting CFU increased. Levofloxacin showed < 0.5-log_10_ change between 10^6^ and 10^7^, but steady decreases in CFU reduction from 10^7^ upwards. Both rifampin and nafcillin acted differently in that antimicrobial efficacy decreased in a step-like fashion between 10^7^ and 10^8^, lacking < 0.5-log_10_ change otherwise. Despite differences in pattern, it is evident that higher starting inoculums cause significant decreases in antimicrobial efficacy.

## 4. Discussion

Percutaneous OI implants are subject to recurring infections attributed to biofilms (Green, 1983, 1984). Bacteria in biofilms have intrinsic antibiotic tolerance, resulting in a noticeable antimicrobial efficacy disparity compared to planktonic bacteria. Antibiotic doses optimized against planktonic bacteria are frequently too low to kill biofilms effectively (Costerton et al., 1999; Olson et al., 2002; la Fuente-Núñez et al., 2013); cells are left alive and more likely to develop antibiotic resistance or serve as a reservoir of infectious agents.

aBL is proposed as an alternative therapy to lessen reliance on antibiotics as the primary defense against infection. It is a localized therapy deemed safe against human skin cells (Joshua et al., 2020). Multiple studies demonstrate aBL efficacy but do not indicate if it is comparable against planktonic and biofilm-dwelling bacteria. Without knowing this pattern, biofilm infections may be undertreated with aBL, resulting in ineffective therapy. To determine if this is a significant concern that must be considered when developing aBL parameters, we studied aBL efficacy against a standard strain in antimicrobial studies, *S. aureus*. This efficacy disparity against *S. aureus* planktonic vs. biofilm bacteria was then compared to levofloxacin, rifampin, and levofloxacin + rifampin to ascertain the potential benefits of aBL over antibiotic regimens.

### 4.1. MBCs for planktonic bacteria

Antibiotic MBCs were orders of magnitude greater than what has been determined in published literature as MBC (and MIC) values are primarily determined against starting inocula of 10^5^-10^6^ CFU/ml (Murillo et al., 2006; Saginur et al., 2006; Trampuz et al., 2007). The tests on different starting concentrations (see Section 2.6) indicate it is likely that our large MBCs were a result of the 10^9^ CFU/ml concentrations utilized throughout this paper. Similarly, Murillo et al. (2006) also used high starting inocula in one their rifampin MBC experiments (~10^8^). Rifampin failed to eradicate bacteria at an MBC that was 512x the MIC. In contrast, most other literature concludes rifampin has excellent antimicrobial efficacy (Coenye and Nelis, 2010; Hu et al., 2015). It has therefore been surmised that low bacterial loads are essential to the success of rifampin-containing regimens (Murillo et al., 2006; Zimmerli and Sendi, 2019).

Nevertheless, our high MBC trend was not limited to rifampin; the levofloxacin MBC obtained in this paper (2.4 mg/ml) was more than 1000x that of common levofloxacin MBC values (1–1.75 μg/ml; Campion et al., 2005; Murillo et al., 2006). A study by Lambert (2000) offers further insight into this observation. Using a variety of antimicrobials, they studied patterns of starting inocula on *S. aureus* MIC values (Lambert, 2000). With increasing inocula, exponential increases in MIC were observed. At an inoculum of 10^8^, the MIC was about 1000x the MIC for 10^5^. Although MIC values were used, the pattern is likely to remain the same in MBCs, supporting the experiments outlined in Section 2.6. Significantly less antimicrobial efficacy was observed for all tested antimicrobials once the CFU concentration increased past 10^8^. Altogether, reported MBC values in this paper were much greater than other published literature, but the experimental data concerning different starting CFU provides some explanation as to why.

Optimally, we would have switched to a lower starting inocula across all experiments, but our methodology was limited in its ability to grow robust and repeatable *S. aureus* biofilms. More relevant bacterial loads (10^5^-10^6^ CFU/coupon) would have required restricting biofilm growth to a 1 mm^2^ area or decreasing biofilm density. Removal of masking around a small 1 mm^2^ area would have disrupted essential biofilm structural components and altered its antimicrobial susceptibility. Additionally, the available irradiated area would have been too small to compare CFU reductions between planktonic and biofilm samples accurately. Biofilm density is also hard to control. We experimentally determined that shortening biofilm growth to 48 h did not remarkably lower the CFU count, and very minimal biofilm formation occurs under 48 h of growth (Rasmussen et al., 2019). Starting CFU concentration was thus maintained at 10^9^ to provide the most accurate and repeatable set-ups for data comparison across all experiments.

### 4.2. MBCs for biofilms

The MBC of levofloxacin against biofilms was ~4x higher than its MBC against planktonic cells. This difference was consistent with general antibiotic trends observed in the literature; bacteria in the biofilm phenotype are more tolerant to antibiotics than planktonic cells (Campion et al., 2005; Stewart, 2015; Mandell et al., 2019; Uruén et al., 2019). The same was true of aBL's biofilm MBC.

Figure 2 indicated that levofloxacin may have limited antimicrobial efficacy against biofilms. The plateau, or lack of further bactericidal action, between 2.4 and 4.8 mg/ml was concerning. Although an increase in CFU reduction was observed after the dose was adjusted to 9.6 mg/ml, another efficacy plateau may be highly probable. It is possible that levofloxacin's killing ability is limited to a maximum of ~3.5-log_10_ CFU and may not be sufficient to completely eradicate biofilms—especially ones with high starting CFU counts. However, closer-spaced data points and a solvent with higher levofloxacin solubility limit would be necessary to validate this conclusion.

In contrast, aBL displayed a linear increase in efficacy against biofilms as exposure time increased. However, aBL revealed the largest antimicrobial efficacy discrepancy between planktonic bacteria and biofilms (see Figure 1), contrary to our hypothesis. The observed outcome was likely a consequence of aBL's delivery as a wavelength. High density biofilms may limit energy transfer to the bottom layer of cells with shorter aBL exposures, preventing ROS production and subsequent cellular death. Yet Figure 2 indicates that density is not an insurmountable barrier. Longer exposure times may have the potential to provide adequate energy to excite porphyrins in the most underlying biofilm cells and generate the required ROS. While confirmation of this would require additional experimentation, extended aBL treatments could provide a means of eradicating biofilm infections even with high starting CFU counts.

Nevertheless, this raises the question of toxicity. Published literature on shorter aBL exposures has concluded that aBL is quite dangerous against retinal cells, but due to limited penetration and greater robustness, skin cells are rarely damaged (Joshua et al., 2020). Unfortunately, there is a lack of long aBL exposure data both *in vitro* and *in vivo*. Evidence of comparable exposure times to those used in this paper (10+ h) is found only in food experiments, such as in the case of eradicating *Salmonella* on pineapple (Ghate et al., 2017). As a result, it is unknown if extended aBL exposures, while effective against biofilms, may have adverse side effects on human cells.

Lastly, while aBL might be more effective than standalone antibiotics such as levofloxacin and rifampin (especially when large CFU reductions are necessary), further data is needed to ascertain whether it can compete as an alternative to combination antibiotic treatments. R/L was the only group with greater antimicrobial efficacy against biofilms than planktonic bacteria in the studied groups. Literature support for this is minimal. While levofloxacin and rifampin are commonly combined for orthopedic infections, such as prosthetic joint infections (PJI), no studies compare their efficacy against planktonic bacteria vs. biofilms (Meléndez-Carmona et al., 2022). Nevertheless, a review of other studies indicates that biofilms are still typically more tolerant to combination antibiotic treatments than planktonic bacteria. A study by Mihailescu et al. (2014) tested planktonic and biofilm forms of MRSA with various combinations of antibiotics, including rifampin. They determined that biofilms were less susceptible than planktonic bacteria. However, it was evident that only rifampin was effective against biofilms as an independent treatment. Therefore, when combined with another antibiotic effective against biofilms, such as levofloxacin, significant antimicrobial efficacy is probable. Additionally, Meléndez-Carmona et al. (2022) performed biofilm studies specifically on a levofloxacin + rifampin combination. They demonstrated that the combination had high efficacy with CDC reactor-grown biofilms (48 h). Nevertheless, they did not test the antibiotics against planktonic bacteria; additional experiments are necessary to understand why our L/R group performed better against biofilms. This observation may be an effect of starting CFU concentration, bacterial strain, or biofilm robustness. Evaluating these factors may reveal possibilities for combining other antibiotics for future orthopedic infection treatment.

However, despite the benefits of combination antibiotics, very high antibiotic concentrations were still required to attain a 3-log_10_ CFU reduction. Increased antimicrobial efficacy may therefore be obtained using aBL with levofloxacin and rifampin. Other studies have indicated aBL works well in synergistic applications. Fila et al. (2017) determined that *Pseudomonas aeruginosa* that was first irradiated with aBL was far more susceptible to subsequent aBL treatment. Woźniak and Grinholc (2022), and a conference presentation by Leanse et al. (2023) demonstrated that aBL with several different antibiotics has synergistic effects. The key to using aBL in OI implant patients might be to use it alongside clinical antibiotics.

### 4.3. Limitations

The high starting CFU count was a notable limitation to the study as antibiotics reached their solubility limit to attain a 3-log_10_ CFU reduction. Although beneficial conclusions were drawn from using aBL and the antibiotics against suspensions with high cellular counts, greater analysis was restricted. In the future, finding a better solvent or repeating the tests with more soluble antibiotics would be helpful. However, other antibiotics may not demonstrate patterns similar to levofloxacin and rifampin. Therefore, exploring new avenues of biofilm growth may also be beneficial. If the CFU count can be lowered without compromising the biofilm structure, excessively high antibiotic concentrations would be unnecessary.

Additionally, only one strain of *S. aureus* was utilized in this study. *S. aureus* ATCC 6538 was selected as it is the standard for disinfectant and antimicrobial testing, and our lab has thorough experience growing and testing it. It presented a benchmark of how *Staphylococcal* strains may act. Nevertheless, Meléndez-Carmona et al. (2022) indicated heterogeneity is present in antimicrobial response among different *S. aureus* strains. It would therefore be highly beneficial to extend the results of this study to different *S. aureus* strains and other bacteria common to orthopedic infections.

## 5. Conclusions

Data from this study indicated that aBL efficacy might depend on *S. aureus* phenotype. Increasing exposure time can potentially overcome aBL's initial limitations and effectively eradicate biofilms. In the case of antibiotics, increasing dosage may be more challenging as toxicity to susceptible organs is problematic. The potential to effectively treat percutaneous OI implant infections with aBL may be to use it in conjunction with antibiotics. However, further data should first be collected to determine aBL efficacy against different bacterial strains and if aBL is safe at longer exposures. Doing so will help progress exploration into aBL's potential as an antibiotic alternative to manage infection at the skin-implant interface of OI implants.

## Data availability statement

The original contributions presented in the study are included in the article/supplementary material, further inquiries can be directed to the corresponding author.

## Author contributions

JO, BI, PP, and DW: conceptualization. JO, RG, AN, and JT: methodology. JO, RG, and DW: validation and writing. JO: formal analysis, writing—original draft preparation, and project administration. DW: resources and supervision. JO and DW: data curation. DW, BI, and PP: funding acquisition. All authors have read and agreed to the published version of the manuscript.
